# Metformin adapts its cellular effects to bioenergetic status in a model of metabolic dysfunction

**DOI:** 10.1038/s41598-018-24017-7

**Published:** 2018-04-04

**Authors:** Christopher Auger, Thibacg Sivayoganathan, Abdikarim Abdullahi, Alexandra Parousis, Bo Wen Pang, Marc G. Jeschke

**Affiliations:** 1 Ross Tilley Burn Centre Sunnybrook Health Sciences Centre Toronto, Ontario, M4N 3M5 Canada; 2 University of Toronto Toronto, Ontario, M5S 1A1 Canada

## Abstract

Thermal injury induces a complex immunometabolic response, characterized by hyperglycemia, extensive inflammation and persistent hypermetabolism. It has been suggested that attenuation of the hypermetabolic response is beneficial for patient wellbeing. To that effect, metformin represents an attractive therapeutic agent, as its effects on glycemia, inflammation and bioenergetics can improve outcomes in burn patients. Therefore, we studied metformin and its effects on mitochondrial bioenergetics in a murine model of thermal injury. We set out to determine the impact of this agent on mitochondrial hypermetabolism (adult mice) and mitochondrial dysfunction (aged mice). Seahorse respirometry complimented by in-gel activity assays were used to elucidate metformin’s cellular mechanism. We found that metformin exerts distinctly different effects, attenuating the hypermetabolic mitochondria of adult mice while significantly improving mitochondrial bioenergetics in the aged mice. Furthermore, we observed that these changes occur both with and without adenosine monophosphate kinase (AMPK) activation, respectively, and analyzed damage markers to provide further context for metformin’s beneficial actions. We suggest that metformin has a dual role following trauma, acting via both AMPK-dependent and independent pathways depending on bioenergetic status. These findings help further our understanding of metformin’s biomolecular effects and support the continued use of this drug in patients.

## Introduction

The systemic response following severe trauma consists of an ‘ebb’ phase within 48 hours, where metabolism, cardiac output and oxygen consumption are all decreased, and a subsequent ‘flow’ phase where these variables gradually increase and plateau^1^. The latter, often referred to as the hypermetabolic period, is characterized by a complex cascade of immune and inflammatory responses which result in increased resting energy expenditure (REE), secretion of catecholamines and stress hormones, lipolysis and hyperglycemia^2,3^. In the short term, this systemic response is necessary to the recovery process, as immunoinflammatory events are required for wound healing, tissue regeneration and the prevention of infections. However, a chronic hypermetabolic phenotype is harmful, as it gives way to the systemic catabolism of adipose and muscle tissues, a loss of lean body mass, increased risk of infections, sepsis and death^4,5^. Conversely, delayed hypermetabolism, wherein the system fails to mount a proper immunoinflammatory response to the traumatic event, may also be devastating to the organism’s wellbeing. For instance, in aging mice and humans, a retarded inflammatory response as evidenced by the delayed appearance of monocytes and lymphocytes, as well as an altered cytokine/chemokine profile underlies the declined would healing ability of the elderly population^6–8^. As such, there is likely an optimal response to injury where hypermetabolism occurs rapidly but is subsequently contained and declines before the onset of systemic dysfunction.

As the metabolic progression following trauma involves variations in oxygen consumption, it’s reasonable to state that changes in mitochondrial dynamics underlie both the hypo- and hypermetabolic phases. This organelle consumes approximately 90% of systemic O_2_ and is responsible for the output of the majority of cellular adenosine triphosphate (ATP), the universal energy currency. The decline and subsequent increase in mitochondrial respiration has been demonstrated in numerous tissues in both animal and human burn studies, implicating this organelle as a driver of the hypermetabolic phenotype^9^. For instance, it has been shown that thermal injury induces the browning of white adipose tissue, a process which increases REE and involves mitochondrial biogenesis and augmented respiration rates^10,11^. As with other immunometabolic events which occur during hypermetabolism, increased mitochondrial bioenergetics are likely essential in the short term to fulfill the energetic needs of the organism. Furthermore, mitochondria-derived reactive oxygen species (ROS) promote the production of pro-inflammatory cytokines such as IL-1β, IL-6 and TNFα^12^. However, overproduction of these moieties leads to cellular damage, and as such, the modulation of mitochondrial activity following thermal injury may limit the noxious effect of this hypermetabolic state^13^. To that end, interventions which regulate mitochondrial bioenergetics following burns have not been thoroughly explored. Insulin, the standard of care for thermal injuries, dramatically improves morbidity and mortality following traumatic events^14^. However, while it mitigates the damage stemming from hypermetabolism, there is no treatment which completely eliminates this phenomenon. Additionally, aggressive insulin therapy has been associated with episodes of hypoglycemia which can worsen patient outcomes^15^. To that effect, we have recently demonstrated in a controlled trial that metformin treatment is equal to insulin in its ability to reduce glucose levels following a severe burn, with the additional benefits of decreased lipolysis, improved insulin resistance and a lower incidence of hypoglycemic episodes^16^.

Metformin’s mechanism of action is attributed primarily to this compound inhibiting complex I of the electron transport chain (ETC). The subsequent lowering of oxidative phosphorylation and consequent drop in ATP levels leads to an increase in the ratio of adenosine monophosphate (AMP) to ATP, thus activating the AMP kinase (AMPK) enzyme^17^. This process then inhibits the expression of hepatic gluconeogenetic genes such as phosphoenolpyruvate carboxykinase and glucose-6-phosphatase, and stimulates the deployment of GLUT4 to the plasma membrane, increasing glucose uptake^18^. This point of view is challenged, however, by findings that metformin can affect cellular pathways and more specifically, mammalian target of rapamycin (mTORC1) signaling independently of AMPK activation^19^. While new mechanisms of action for this biguanide are progressively uncovered, its interest by other fields continues to grow, and metformin is thought to have therapeutic value in the domains of cancer, cardiovascular disorders and aging^20^. The use of this drug beyond the scope of diabetes implores an extensive examination of its precise molecular effects.

**Figure 1:**
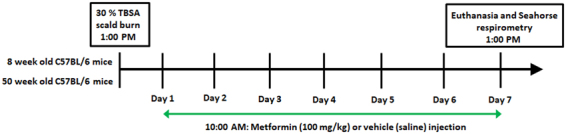
Timeline and study design. Adult (8 week old) and aged (50 week old) mice were divided into the following groups: control; control + metformin; burn; burn + metformin. Mice not receiving metformin (100 mg/kg) were given saline via intraperitoneal injection. TBSA: total body surface area.

The aim of this study was to demonstrate and pharmacologically correct two disparate types of mitochondrial dysfunction, one where there is a compensatory recovery and the organelle is hyperactive (adult mice) and one where mitochondrial recovery is delayed and bioenergetics are diminished (aged mice). To accomplish this, mitochondrial dynamics in livers from both adult and aged mice subjected to a 30% total body surface area (TBSA) burn were analyzed in order to gauge age-dependent variations in bioenergetics as well as the response to an intervention with metformin (Fig. 1). As a central metabolic organ and one of the primary tissues contributing to post-burn hypermetabolism, the liver was chosen for this study^2^. Since treatment with metformin has been shown to impact mitochondrial function in other models, we hypothesized that metformin could also provide therapeutic benefits following burn injury via its actions on mitochondrial dynamics. Other parameters, such as protein oxidation, nitrosylation of tyrosine residues and circulating mitochondrial DNA (mtDNA) were also analyzed, to further elucidate metformin’s biological effects.

## Results

### Metformin treatment suppresses mitochondrial activity post-burn in adult mice

**Figure 2:**
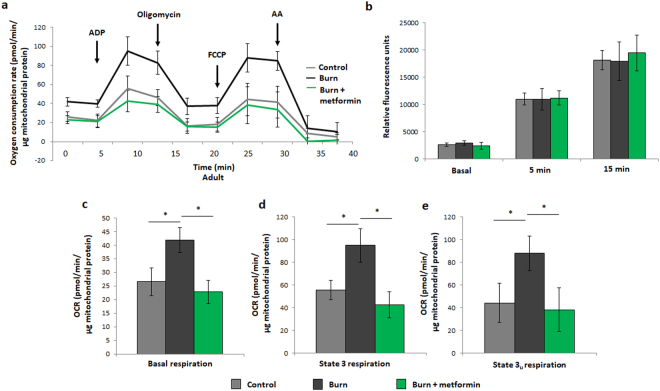
Seahorse XF96 respirometry analysis of adult mice at day 7 post injury. (**a**) Respiration profiles of liver mitochondria from control mice (grey), burned mice (black) and burned mice with metformin treatment (100 mg/kg; green). (**b**) Production of reactive oxygen species as measured by oxidation of 2,7-dichlorodihydrofluorescein-diacetate (DCFDA) in isolated mitochondria given substrate (5 mM pyruvate, 3 mM malate, 4 mM ADP); λ = 495 nm, λ’ = 529 nm. Basal (**c**), state 3 (**d**) and state 3 u (**e**) respiration parameters in isolated mitochondria as measured via Seahorse XF96 extracellular flux assays. : control (n = 5); ■: burn (n = 5); : burn + metformin treatment (n = 6). Values are presented as mean ± standard deviation. *p ≤ 0.05.

**Figure 3:**
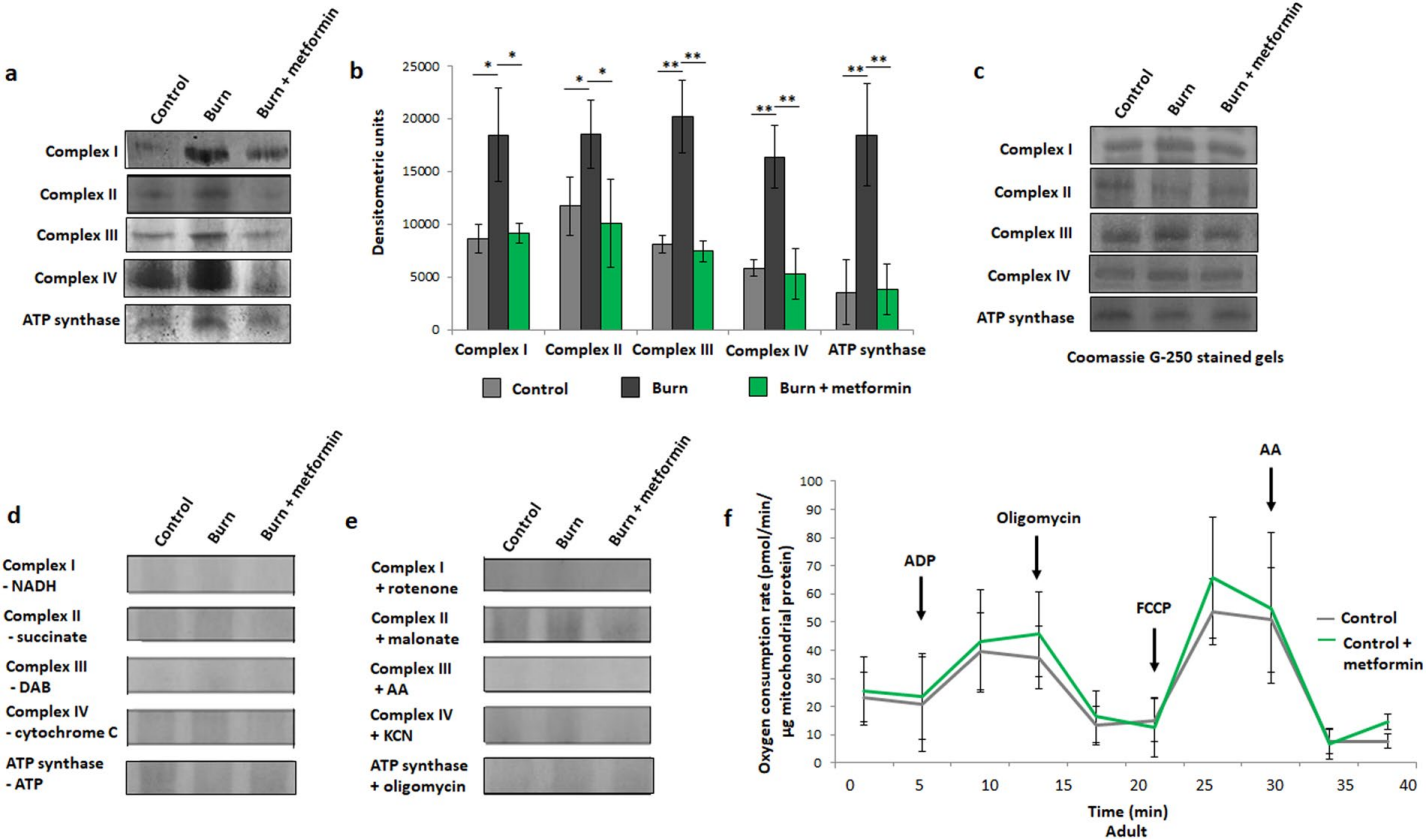
In-gel activity assays for electron transport chain (ETC) complexes from adult mice. (**a**) Representative images of complexes I-IV and ATP synthase of the ETC, assayed following blue native polyacrylamide gel electrophoresis of liver mitochondrial homogenates to demonstrate the effect of metformin in injured adult animals. (**b**) Densitometric measurements of in-gel activity assay bands using ImageJ for Windows. (**c**) Bands selected from the gel following staining with Coomassie Blue G-250 served as a loading control. In-gel activity assays from panel **a** were performed in the absence of key reaction mixture substrates (**d**) or in the presence of inhibitors (**e**) to confirm band specificity. (**f**) Respiration profiles of liver mitochondria from adult control mice (grey; n = 9) and control mice given metformin (green; n = 9). : control (n = 5); ■: burn (n = 5); : burn + metformin treatment (n = 5). Values are presented as mean ± standard deviation. *p ≤ 0.05; **p ≤ 0.01.

As previously described, the compensatory recovery of mitochondrial bioenergetics in burned adult mice was observed in freshly-isolated liver mitochondria^9^. While the burned mice display a significantly higher respiration profile than their control counterparts, metformin treatment post-burn appears to return oxygen consumption rates (OCR) to control levels (Fig. 2a). Interestingly, these variances in respiration do not appear to affect ROS production by isolated mitochondria, as analyzed by DCFDA oxidation (Fig. 2b). Relative to burn alone, major respiratory parameters are indeed decreased with metformin treatment upon further analysis of the respiration profiles (Fig. 2c–e). To confirm this finding, isolated mitochondria were prepared for native PAGE studies to measure the in-gel enzymatic activity of the ETC complexes as well as ATP synthase. All five components of oxidative phosphorylation displayed lower activity with metformin treatment when compared to burn alone (Fig. 3a), and this result was ascertained using densitometric software (Fig. 3b). Owing to a lack of loading control (e.g., GAPDH in Western blots) for in-gel activity assays, gels were stained with Coomassie to verify equal loading (Fig. 3c). To confirm band specificity, negative controls where key substrates were omitted from the reaction mixture (Fig. 3d) or inhibitory agents were added to the reaction (Fig. 3e) were performed. Here, the absence of bands served as evidence that the reactions in Fig. 3a were indeed the enzymes of interest. Furthermore, this respiratory lowering effect of metformin is dependent on the pathological state induced by the burn injury, as treating unburned control mice with the biguanide did not induce this outcome (Fig. 3f). While this may seem to counteract the hypothesis that metformin acts by inhibiting complex I, it’s important to note that this study is observing the effects of chronic and not acute metformin treatment and that the concentration of this agent given (100 mg/kg per day) is low relative to other studies (up to 500 mg/kg)^20^.

**Figure 4:**
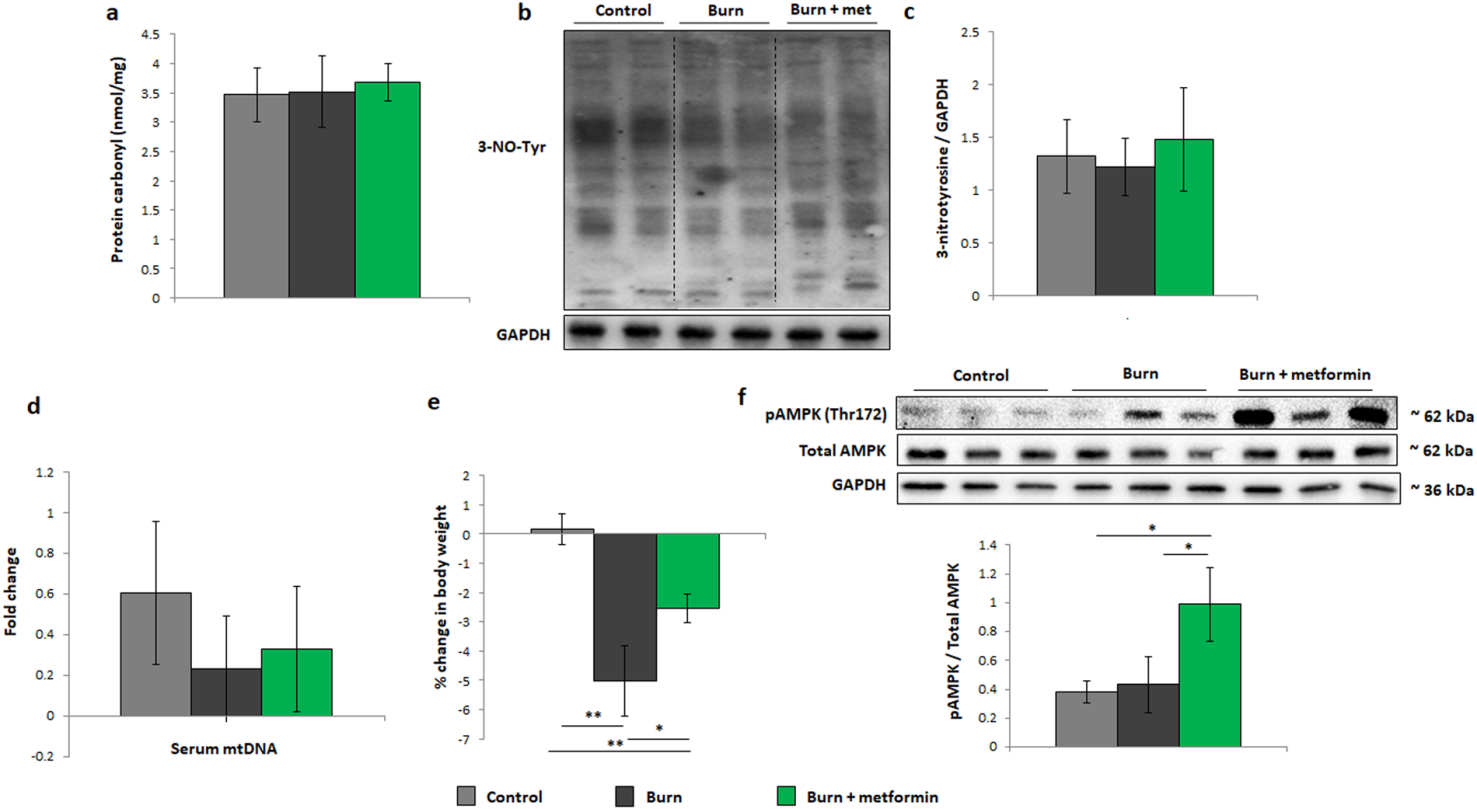
Damage markers and metformin signaling in adult mice. (**a**) Protein oxidation of liver homogenates as measured by derivatization with 2,4-dinitrophenylhydrazine (DNPH). (**b**) Representative cropped Western blot of 3-nitrotyrosine residues and GADPH in liver homogenates from adult mice. (**c**) Densitometric measurements of 3-nitrotyrosine blots from control (n = 4), burn (n = 5) and burn + metformin-treated mice (n = 5). (**d**) Circulating plasma mitochondrial DNA from control, injured and treated mice. (**e**) Changes in body weight between day 1 and day 7 in all adult cohorts as expressed as % of the original mass. (**f**) Representative Western blots of phosphorylated adenosine monophosphate-activated protein kinase (pAMPK), total AMPK, and glyceraldehyde 3-phosphate dehydrogenase (GAPDH). Densitometry was performed to assess the activation of AMPK via its phosphorylation. : control (n = 5); ■: burn (n = 5); : burn + metformin treatment (n = 5). Values are presented as mean ± standard deviation. *p ≤ 0.05; **p ≤ 0.01.

To determine whether burn injury with or without metformin treatment has an effect on the redox environment, levels of protein carbonyl (Fig. 4a) and nitrosylated tyrosine residues (Fig. 4b,c) were analyzed. The latter is a marker for the formation of peroxynitrite, a potent oxidizing and nitrating agent formed by the coupling of superoxide and nitric oxide intracellularly^21^. No significant changes were observed in oxidation markers in either the burn or burn treated with metformin groups. Circulating mtDNA, a damage-associated molecular pattern (DAMP) which can initiate an inflammatory response was also measured but barely detectable in adult plasma in either group (Fig. 4d)^9,22^. As one of the primary outcomes of burn injury and the hypermetabolic phenotype is the loss of body mass, we compared the weight loss of the three groups, and show that metformin-treated mice lose mass to a lesser extent than untreated animals post-burn (Fig. 4e). In order to delineate metformin’s signaling mechanism, Western blotting was performed on phosphorylated (Thr172) and total AMPK. Indeed, the increase in the phosphorylated variant of the enzyme would indicate that metformin treatment in these mice is activating this oft-reported pathway (Fig. 4f). Taken together, these findings in adult mice suggest that metformin reduces the burn-induced increase in respiration and activates AMPK while having no effect on damage markers which were not significantly altered by the injury at this time point.

### Metformin treatment increases mitochondrial activity post-burn in aged mice

**Figure 5:**
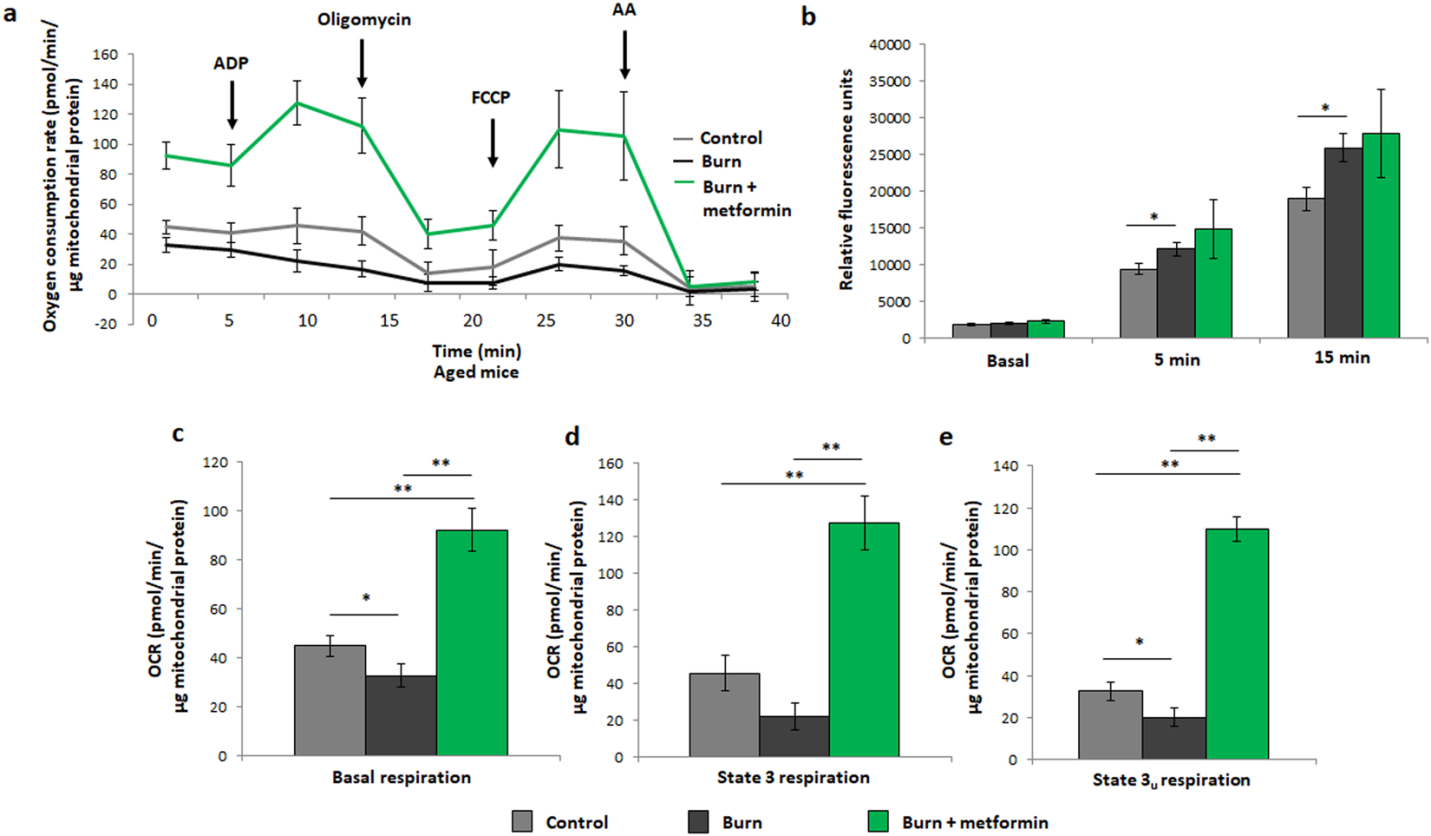
Seahorse XF96 respirometry analysis of aged mice at day 7 post injury. (**a**) Respiration profiles of liver mitochondria from control mice (grey), burned mice (black) and burned mice with metformin treatment (100 mg/kg; green). (**b**) Production of reactive oxygen species as measured by oxidation of DCFDA in isolated mitochondria given substrate (5 mM pyruvate, 3 mM malate, 4 mM ADP); λ = 495 nm, λ’ = 529 nm. Basal (**c**), state 3 (**d**) and state 3 u (**e)** respiration parameters in isolated mitochondria as measured via Seahorse XF96 extracellular flux assays. : control (n = 5); ■: burn (n = 5); : burn + metformin treatment (n = 6). Values are presented as mean ± standard deviation. *p ≤ 0.05; **p ≤ 0.01.

**Figure 6:**
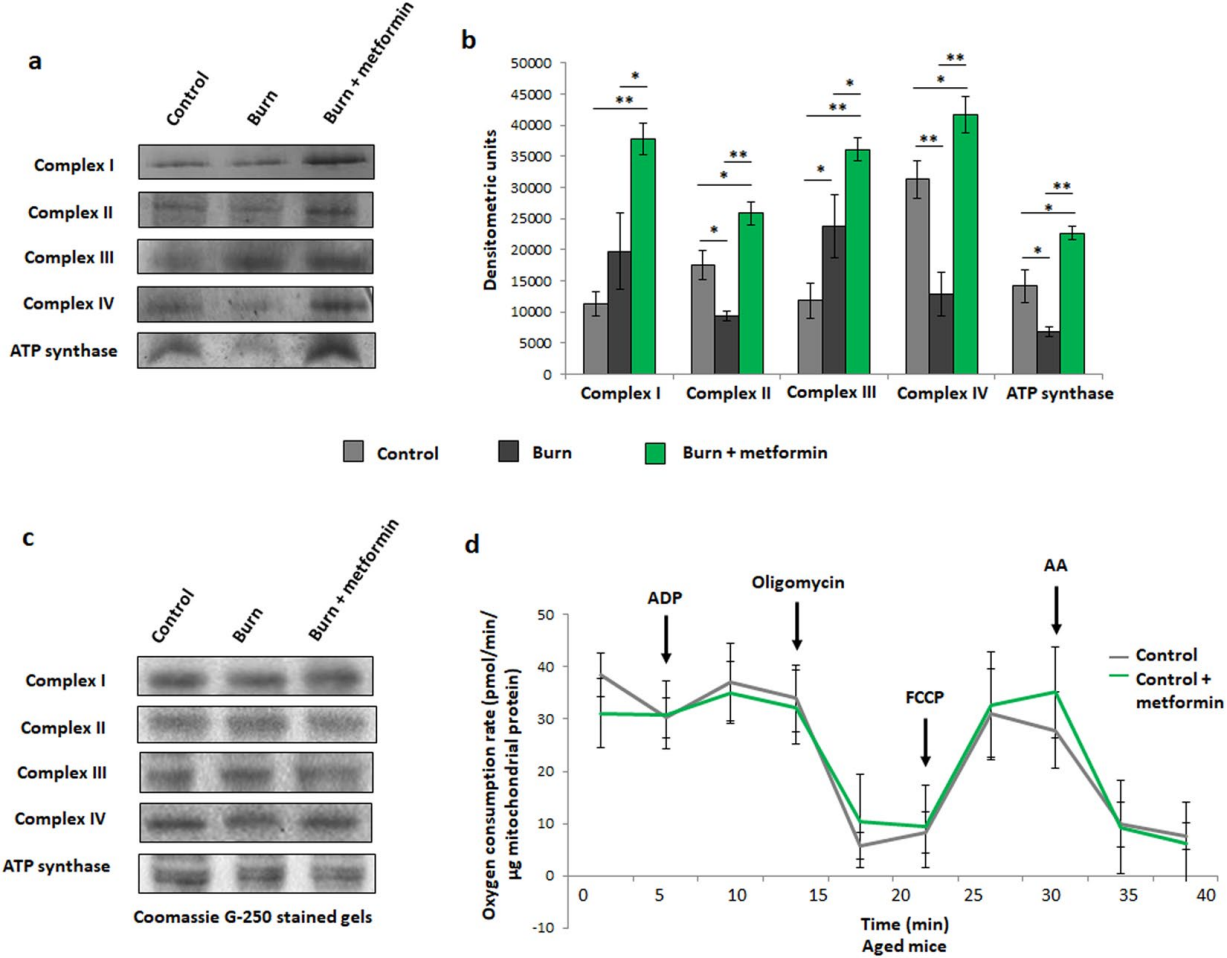
Enzyme activity assays and control Seahorse XF96 curves from aged mice. (**a**) Representative images of complexes I-IV and ATP synthase of the ETC, assayed following blue native polyacrylamide gel electrophoresis of liver mitochondrial homogenates to demonstrate the effect of metformin in injured aged animals. (**b**) Densitometric measurements of in-gel activity assay bands using ImageJ for Windows. (**c**) Bands selected from the gel following staining with Coomassie Blue G-250 served as a loading control. (**d**) Respiration profiles of liver mitochondria from aged control mice (grey) and control mice given metformin (green). : control (n = 5); ■: burn (n = 5); : burn + metformin treatment (n = 6). Values are presented as mean ± standard deviation. *p ≤ 0.05; **p ≤ 0.01.

While the respiration profile of adult burned mice shows a compensatory recovery at 7 days post-burn, the aged cohort fails to recover at this time point, as indicated by the lowest OCR curve reported (Fig. 5a). As metformin treatment inhibits complex I and was shown to lower respiration relative to the adult burn group alone (Fig. 2a), we anticipated a further drop in OCR upon treatment with this therapeutic agent. Interestingly, a 7 day metformin treatment significantly up-regulated respiration in the aged mice relative to burn alone, facilitating the recovery of hepatic bioenergetics post-burn (Fig. 5a). In the burn group, isolated mitochondria produced significantly more ROS, perhaps indicative of damage to and uncoupling of the electron transport chain, while the metformin-treated cohort showed no significant difference (Fig. 5b). Further analyses of the Seahorse XF96 results confirm metformin’s ability to increase the major respiratory parameters relative to the aged burn group alone (Fig. 5c–e). As with the adult mice, in-gel activity assays were performed as a complimentary analysis to respiration data. Here we can see that, while burn injury significantly decreases the activities of complexes II, IV and ATP synthase, the first and third members of the ETC appear to have increased activity following burn (Fig. 6a). Densitometric analyses on the in-gel activity images show that this is indeed the case, with increases to complex III being significant and complex I bordering on significance (Fig. 6b; p = 0.057). As these two protein complexes are known as the principle sites of superoxide generation within the ETC, their sustained activity post-burn explains the increased ROS production detected by DCFDA (Fig. 5b)^23^. Metformin treatment increased the activity of all complexes significantly (Fig. 6a). Equal loading was confirmed via Coomassie staining (Fig. 6c) and negative controls to confirm enzyme specificity were performed as with the adult mice (data not shown). As with the adult animals, chronic metformin treatment in aged control mice does not promote changes in bioenergetics, thus demonstrating that metformin’s ability to enhance mitochondrial activity is contingent on the pathological state induced by the burn injury (Fig. 6d).

**Figure 7:**
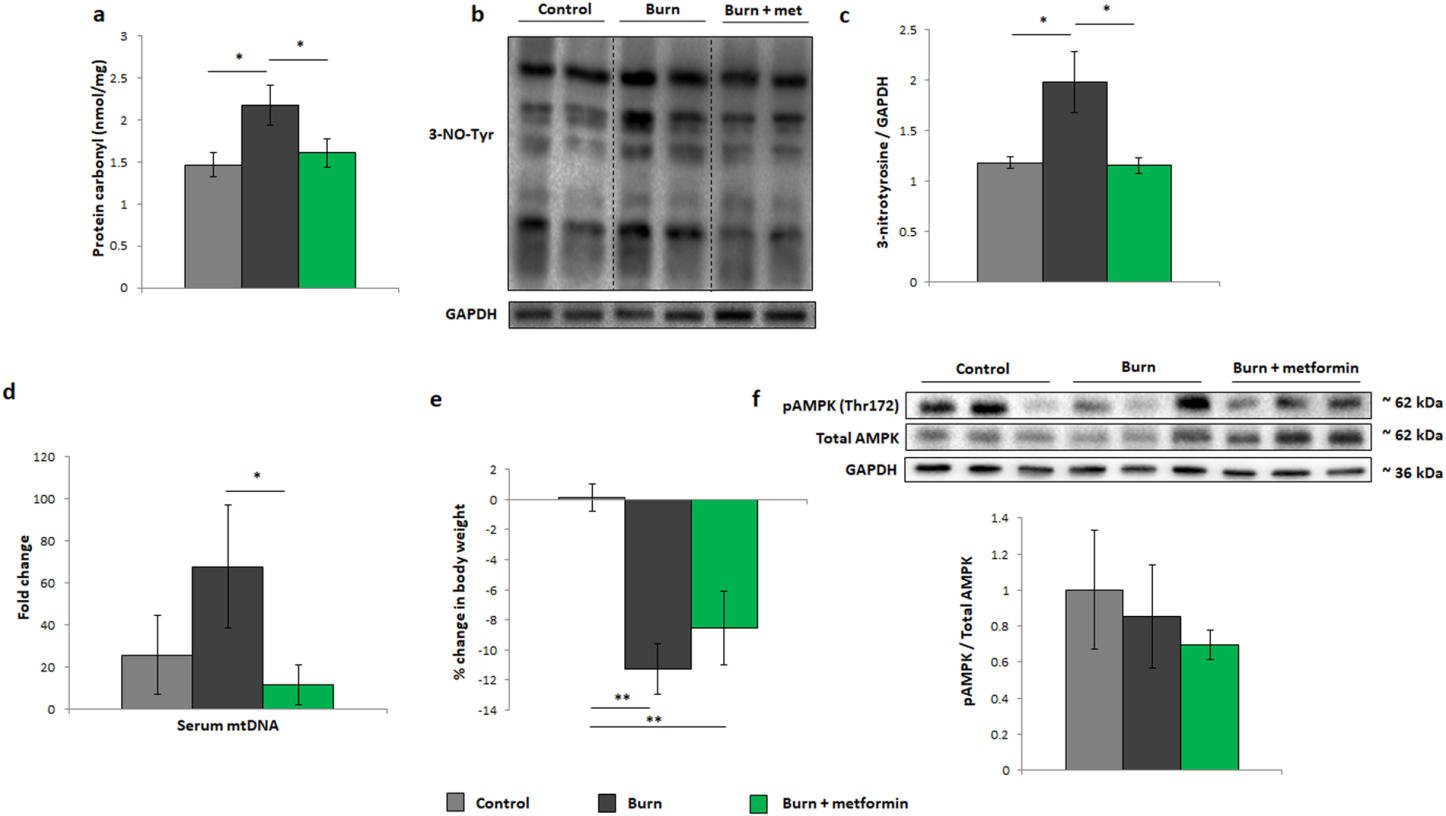
Damage markers and metformin signaling in aged mice. (**a**) Protein oxidation of liver homogenates as measured by derivatization with DNPH. (**b**) Representative cropped Western blot of 3-nitrotyrosine residues and GAPDH in liver homogenates from aged mice. (**c**) Densitometric measurements of 3-nitrotyrosine blots from control (n = 4), burn (n = 5) and burn + metformin-treated mice (n = 5). (**d**) Circulating plasma mitochondrial DNA from control, injured and treated mice. (**e**) Changes in body weight between day 1 and day 7 in all aged cohorts as expressed as % of the original mass. (**f**) Representative Western blots of pAMPK, total AMPK and GAPDH. Densitometry was performed to assess the activation of AMPK via its phosphorylation. : control (n = 5); ■: burn (n = 5); : burn + metformin treatment (n = 5). Values are presented as mean ± standard deviation. *p ≤ 0.05.

To elucidate metformin’s potential hepatic benefits in the aged mice, oxidized proteins (Fig. 7a) and nitrosylated tyrosine residues (Fig. 7b,c) were analyzed. Although these moieties increase in the burned cohort, metformin treatment significantly lowers the amount of both, indicative of this agent’s ability to decrease oxidative damage. Furthermore, serum levels of mtDNA were analyzed, and metformin treatment does lower the concentrations of this DAMP in injured animals (Fig. 7d). Despite these changes, metformin treatment does not significantly improve the loss of body mass in the aged animals (Fig. 7e). As such, a longer treatment in this cohort may be necessary to slow the systemic catabolism induced by burn trauma. To elucidate the signaling mechanism of metformin post-burn, levels of phosphorylated (Thr172) and total AMPK were measured by Western blot. While in the adult group, metformin activated AMPK following the inhibition of ETC activity, the aged group showed no significant difference in AMPK signaling (Fig. 7f). Therefore, it appears as though a regimen of metformin in aged mice increases respiration, having no effect on AMPK while also reducing the observed damage markers that are significantly altered by trauma in this age group.

## Discussion

Here we present a murine trauma model where, depending on the age of the animal, two forms of mitochondrial dysfunction are made evident and corrected by metformin treatment. To our knowledge, this is the first reported example which showcases the dual role played by this agent in the regulation of mitochondrial bioenergetics and the involvement of AMPK signaling in the individual outcomes. Variations in mitochondrial respiration following a traumatic injury have profound implications. Hypermetabolism, a heightened REE, is accompanied by long-term increases in OCR and characterized by systemic catabolism, loss of lean body mass and increased susceptibility to infection^24^. Conversely, persistent hypometabolism, demarcated by the delayed recovery of mitochondrial respiration following trauma, could lead to failure of biological systems to fulfill their energetic needs^25,26^. The data presented herein highlight both of these mitochondrial abnormalities, with the adult mice exhibiting a hypermetabolic phenotype at 1 week following thermal trauma, and aged mice failing to exhibit a compensatory recovery by this time point. Although chronic hypermetabolism is associated with an abundance of systemic complications, we and others postulate that a short-term increase in metabolic flux is beneficial following trauma^27^. Indeed, we have recently shown in burn patients that the elderly have delayed hypermetabolism versus their younger counterparts, a phenomenon which may underlie their increased mortality following severe burns^26^. The inability of mitochondria to rebound from the initial trauma and consequently, a decrease in ATP production, are detrimental to the recovery process^27^.

Metformin is an attractive therapeutic option during hypermetabolic states not only for its ability to limit stress-induced hyperglycemia but also to suppress the increased oxygen consumption via the inhibition of complex I. To that end, there is a debate on this biguanide’s intracellular mechanism of action, particularly with regards to its effects on mitochondrial respiration. While many studies report a diminishment of ETC activity upon metformin treatment, there are others which report an increase in mitochondrial energy formation^28–31^. Using a murine model of thermal trauma, we show here that administration of metformin can both lower and increase mitochondrial respiration, depending on the state of bioenergetics during treatment. In the context of burns, this appears to be an ideal therapeutic intervention, as the hypermetabolic state in the adult population which can persist for months to years post-trauma is undesirable and decreasing the REE via pharmaceutical regulation is linked to better outcomes^32^. On the other hand, a notable delay in hypermetabolism as demonstrated by the aged cohort is also devastating, and metformin’s ability to bolster mitochondrial energy production in this population may be beneficial, although this remains to be seen. The lowering of protein nitrosylation and circulating mtDNA in serum support this notion. However, metformin does not slow the loss of mass in this cohort and as such, further studies are required to elucidate the benefits of this pharmacological intervention. From the data presented herein, it appears as though metformin’s signaling mechanism (AMPK dependent/independent) may be determined by mitochondrial status. Logically, metformin’s inhibition of the electron transport chain activity in the adult group would alter the AMP/ATP ratio, thus activating AMPK. However, in the aged group where mitochondria fail to recover, metformin’s actions proceed independently of this enzyme, and mitochondrial respiration is increased via an unknown mechanism. This finding is in agreement with previous studies which have shown that an increase in respiratory parameters induced by metformin treatment occurs in the absence of AMPK signaling^28^. Moreover, others have shown that AMPK-dependent repair mechanisms become less competent with age, perhaps explaining why metformin fails to activate this pathway in the older cohort^33^.

An important limitation of the work presented here is the age of the mice (50 weeks), which is not geriatric, but was chosen as a model for the physiological processes which underlie ageing without the high rates of mortality that would result from a traumatic burn in older mice. Indeed, we and others have shown that in mice aged to 50 weeks, mitochondrial processes begin to decline, and we hypothesize that the mitochondrial dysfunction and damage markers reported here would be more profound in geriatric animals^27,34,35^. This study elaborates on the hepatic mitochondrial abnormalities which ensue following a burn injury, a severe form of trauma with dire metabolic consequences. It appears as though, depending on the age of the organism, two vastly different outcomes are possible; one where mitochondrial respiration is up-regulated and a contributing factor to the hypermetabolic phenotype, and one where mitochondrial bioenergetics fail to rebound from the traumatic event, resulting in the ineffective generation of energy. Interestingly, metformin treatment appears to correct both types of mitochondrial dysfunction, suggesting a dual role for this therapeutic agent at the organellar level. Given the scope of illnesses where metformin administration is advised, a greater understanding of the biomolecular pathways targeted by this drug is required. To elaborate on its potentially beneficial effects, we also demonstrate this biguanide’s ability to reduce protein oxidation/nitrosylation and the circulation of inflammatory mtDNA. Thus, the data presented herein further elucidates metformin’s role in the regulation of mitochondrial dynamics following severe burns and supports the continued use of this agent in the treatment of trauma survivors.

## Methods

### Mouse model

Male C57BL/6 mice (Jackson Laboratory) were housed at ambient temperature and cared for in accordance with the Guide for the Care and Use of Laboratory Animals. All procedures performed in this study were approved by the Sunnybrook Research Institute Animal Care Committee. Adult mice were 8 weeks of age, while the aged mice selected for this study were 50 weeks. Mice were anesthetized by inhalation of 3–5% isoflurane and given a 0.1 mg/kg intraperitoneal (i.p.) injection of buprenorphine. Approximately 40% of the dorsum and ventral regions were shaved with electrical clippers and ~ 1cc of lactated ringers solution was injected under the skin along the spinal column as well as i.p. Full thickness third degree burns covering 30% of the TBSA were achieved by immersing the back of the mice at 98 °C for 10 s and the ventral region for 2 s to avoid organ damage. Burned mice were subsequently housed individually in sterile cages and fed ad libitum until sacrifice. Select mice were treated with a daily i.p. injection of metformin (100 mg/kg). All injured rodents were scored by both certified veterinarians and laboratory staff daily to minimize animal pain and distress, and the analgesic buprenorphine was administered to mitigate pain when indicated. Mice were sacrificed at 7 days post-thermal injury. Livers were harvested for further analysis and blood collected following cardiac puncture. Sham mice (control) underwent identical experimental procedures, with the exception of the burn injury. Mice not receiving metformin were given vehicle (saline) injections.

### Mitochondrial isolation and respirometry

Freshly-excised livers were minced in 10 volumes of mitochondrial isolation buffer (MHSE + BSA; 210 mM mannitol, 70 mM sucrose, 5 mM HEPES, 1 mM EGTA, 0.5% (w/v) fatty acid-free BSA, pH 7.2) as described^36^. The tissue was then homogenized using a Teflon glass homogenizer with 6 strokes and filtered through three layers of cheesecloth. Mitochondria were isolated via differential centrifugation. Briefly, the homogenate was centrifuged at 600 g for 10 min and the supernatant decanted into a new tube. This fraction was centrifuged at 9000 g for 10 min to afford a mitochondrial pellet. The resultant supernatant which contains soluble proteins was subjected to a BCA protein assay (Thermo Scientific) and set aside for other assays. A clear lipid layer surrounding the mitochondria was carefully aspirated and the pellet re-suspended in 200 uL of MHSE + BSA. A second centrifugation to purify mitochondria was performed as per the protocol by Rogers *et al*.^37^. BCA assays were performed to gauge protein concentrations. Mitochondria were pelleted in a 96-well plate via centrifugation at 2000 g for 20 min at 4 °C and bioenergetics assessed using a Seahorse XF96 analyzer (Agilent Technologies). Mitochondrial respiration in a coupled state (10 μg/well) was measured in mitochondrial assay solution (MAS; 220 mM mannitol, 70 mM sucrose, 10 mM KH_2_PO_4_, 5 mM MgCl_2_, 2 mM HEPES, 1 mM EGTA and 0.2% (w/v) fatty acid-free BSA, pH 7.2 at 37 °C) containing succinate as a substrate (10 mM) and rotenone (2 μM). State 3 respiration (phosphorylating respiration) was triggered via the injection of 4 mM ADP. State 4o respiration was assessed by the addition of 2.5 μg/mL oligomycin, while maximal uncoupler-stimulated respiration was observed following the injection of 4 μM carbonyl cyanide 4-(trifluoromethoxy)phenylhydrazone (FCCP). Antimycin A (4 μM), a Complex III inhibitor, was added at the end of the experiment to inhibit mitochondrial respiration, as described^37^. Liver mitochondria from 5 control and 5 burned mice were included in each Seahorse analysis, and every sample analyzed in triplicate. The Seahorse XF Wave software was used to group the respiration data from separate mice into a single representative curve and data was normalized to mitochondrial protein content.

### Mitochondrial ROS production

Liver mitochondria isolated as described above were tested for ROS emission via the fluorogenic dye 2,7-dichlorodihydrofluorescein-diacetate (DCFDA). Freshly isolated mitochondria were diluted to 0.5 mg/ml in reaction buffer (20 mM Tris base, 125 mM sucrose, 2 mM MgCl_2_, 4 mM KH_2_PO_4_, 1 mM EDTA) in a 96 well plate. Twenty μM DCFDA was added and mitochondria were warmed to 37 °C for 5 min, at which point baseline ROS measurements were recorded (λ_ex_ = 495 nm; λ_em_ = 529 nm). Mitochondria were subsequently treated with 5 mM pyruvate, 3 mM malate and 4 mM ADP to drive respiration and DCFDA measurements were taken at 5 min and 15 min (Synergy H4 Hybrid Reader; BioTek). Data were normalized to background fluorescence. All mitochondrial experiments were performed within 4 h of the isolation.

### Electrophoresis and in-gel activity assays

Blue native (BN) polyacrylamide gel electropohoresis (PAGE) was performed as described^36,38^. Briefly, mitochondrial fractions were isolated and prepared in a non-denaturing buffer (50 mM Bis-Tris, 500 mM ε-aminocaproic acid, pH 7.0, 4 °C) at a concentration of 4 µg/µL. Ten percent n-dodecyl β-D-maltoside was added to mitochondrial fractions to solubilize membrane bound proteins. A 2–10% or 4–16% gradient gel was prepared (Bio-Rad MiniProteanTM 2 system) using 1 mm spacers to ensure optimal protein separation. Sixty micrograms of protein were loaded into each well and electrophoresed under native conditions in anode buffer (50 mM Bis-Tris, pH 7 at 4 °C) at 80 V to ensure proper stacking, then at 300 V for proper migration through the gel, ensuring the current does not surpass 25 mA. The blue cathode buffer (50 mM Tricine, 15 min Bis-Tris, 0.02% (w/v) Coomassie G-250, pH 7 at 4 °C) was utilized to help visualize the running front and was exchanged to a colourless cathode buffer (50 mM Tricine, 15 mM Bis-Tris, pH 7 at 4 °C) when the running front was halfway through the gel. Upon completion, the gel was equilibrated in reaction buffer (25 mM Tris-HCl, 5 mM MgCl_2_, pH 7.4) for 15–30 min. Complex I was detected by the addition of 5 mM KCN, 1 mM NADH and 0.4 mg/mL iodonitrotetrazolium chloride. Analysis of complex II was achieved with a reaction mixture containing 20 mM succinate, 0.2 mM phenazine methosulfate and 25 mg nitrotetrazolium blue in 10 mL of 5 mM Tris/HCl, pH 7.4. Complex III activity was assessed with 5 mg diaminobenzidine (DAB) in 10 mL of 50 mM sodium phosphate, pH 7.2. Complex IV was assayed by the addition of 10 mg/mL of DAB, 10 mg/mL cytochrome C, and 562.5 mg/mL of sucrose. A reaction mixture containing 35 mM Tris, 270 mM glycine, 14 mM MgCl_2_, 0.2% Pb(NO_3_)_2_ and 8 mM ATP permitted the visualization of ATP synthase activity. Negative controls for the activity of ETC complexes I-IV and ATP synthase lacked NADH, succinate, DAB, cytochrome C and ATP, respectively. Inhibition of their respective activities was accomplished via the addition of 10 µM rotenone, 5 mM malonate, 40 µM antimycin A, 5 mM KCN or 5 µg/mL oligomycin to the reaction mixtures. Reactions were stopped using a destaining solution (40% methanol, 10% glacial acetic acid) once the activity bands had reached the desired intensity. Acetic acid was omitted for the ATP synthase reaction, as the acid would dissolve the lead phosphate precipitate. Gels were imaged colorometrically using a Bio-Rad Chemidoc Imaging System with the exception of ATP synthase which required fluorescent imaging settings to capture the lead phosphate precipitate. These images were then converted to gray scale and densitometry was performed using Image J for Windows.

### Oxidized protein assays

Total protein carbonyl content was assessed by first mixing 1 mg of soluble protein with 0.2% (w/v) 2,4-dinitrophenylhydrazine (DNPH). After 60 mins of incubation at room temperature, 200 μL of 50% (w/v) TCA was added to each sample. The resulting precipitate was removed by centrifugation at 18,000 g for 10 min. The pellet was washed 3 times with 10% (w/v) trichloroacetic acid followed by centrifugation at 18,000 g for 10 min. The protein pellet was then washed thrice with a solution of ethylacetate:ethanol (1:1). The pellet was dissolved in 600 μL of 6 M guanidine and the absorbance was measured at 370 nm (ε = 21.5 M^−1^ cm^−1^).

### Immunoblot experiments

To characterize the expression of AMPK, pAMPK, GAPDH and nitrosylated tyrosine (NO-Tyr) residues, Western blots were performed. Following SDS-PAGE of liver lysate (30 ug) from control and burned mice, the proteins were transferred using a Trans-Blot Turbo Transfer System (Bio-Rad). Upon completion, the nonspecific binding sites on the membrane were blocked using 5% (w/v) non-fat skim milk for 1 h. The membrane was then washed twice in TTBS (12.1 g Tris, 40 g NaCl, 2% Tween 20; pH 7.6 at 4 °C) for 5 min. Blots were incubated overnight with the primary antibody [1:1000 AMPK (Cell Signaling; #2532 S); 1:1000 pAMPK (Thr172; Cell Signaling; #2531 S); 1:5000 GAPDH (Abcam; ab8245); 1:1000 NO-Tyr (Abcam; ab7048)]. The membrane was washed twice in TTBS for 5 min, followed by incubation with secondary antibody, which consisted of horseradish peroxidase-conjugated anti-rabbit or anti-mouse antibodies (1:3000) for 1 h. The membrane was washed in TTBS twice for a period of 5 min and detection of the desired protein was achieved with a BioRad ChemiDoc Imaging System. All antibodies were obtained from Abcam. SuperSignal West Pico Chemiluminescent Substrate was a product of Thermo Scientific Inc. Densitometry was performed using Image J for Windows.

### Quantification of plasma mtDNA

Fresh blood was collected in EDTA tubes and centrifuged for 20 min at 3000 rpm. Mitochondrial DNA was quantified with real-time qPCR using a StepONE Plus PCR System (Applied Biosystems, California, USA) and Biorad SsoAdvanced Universal SYBR^®^ Green Supermix (Bio-Rad, California, USA) according to manufacturer’s directions. Primers were selected that amplify the NADH dehydrogenase subunit 1 motif on the mitochondrial genome. Primers used were as follows; *NAD*1 (F′: CCC ATT CGC GTT ATT CTT, R′; AAG TTG ATC GTA ACG GAA GC). Optimization was performed to determine a 1:20 dilution ratio of plasma in nuclease free sterile water and 2 ul of starting material was used per reaction. Relative ND1 copy number was calculated based on the threshold cycle. All samples were run in duplicate, simultaneously with negative controls.

### Statistical analysis

Data were expressed as means+/− standard deviation. Statistical correlations of the data were checked for significance using the Student t test or one-way ANOVA followed by Tukey post-hoc (p ≤ 0.05). Five control mice and at minimum five burned mice were included in each analysis.
